# A temporal proteome dynamics study reveals the molecular basis of induced phenotypic resistance in *Mycobacterium smegmatis* at sub-lethal rifampicin concentrations

**DOI:** 10.1038/srep43858

**Published:** 2017-03-06

**Authors:** Alexander D. Giddey, Elise de Kock, Kehilwe C. Nakedi, Shaun Garnett, Andrew J. M. Nel, Nelson C. Soares, Jonathan M. Blackburn

**Affiliations:** 1 Department of Integrative Biomedical Sciences, University of Cape Town, South Africa; 2 Institute of Infectious Disease & Molecular Medicine, University of Cape Town, South Africa

## Abstract

In the last 40 years only one new antitubercular drug has been approved, whilst resistance to current drugs, including rifampicin, is spreading. Here, we used the model organism *Mycobacterium smegmatis* to study mechanisms of phenotypic mycobacterial resistance, employing quantitative mass spectrometry-based proteomics to investigate the temporal effects of sub-lethal concentrations of rifampicin on the mycobacterial proteome at time-points corresponding to early response, onset of bacteriostasis and early recovery. Across 18 samples, a total of 3,218 proteins were identified from 31,846 distinct peptides averaging 16,250 identified peptides per sample. We found evidence that two component signal transduction systems (e.g. MprA/MprB) play a major role during initial mycobacterial adaptive responses to sub-lethal rifampicin and that, after dampening an initial SOS response, the bacteria supress the DevR (DosR) regulon and also upregulate their transcriptional and translational machineries. Furthermore, we found a co-ordinated dysregulation in haeme and mycobactin synthesis. Finally, gradual upregulation of the *M. smegmatis*-specific rifampin ADP-ribosyl transferase was observed which, together with upregulation of transcriptional and translational machinery, likely explains recovery of normal growth. Overall, our data indicates that in mycobacteria, sub-lethal rifampicin triggers a concerted phenotypic response that contrasts significantly with that observed at higher antimicrobial doses.

*Mycobacterium tuberculosis* is the causative agent of tuberculosis disease (TB), which accounts for ~1.5 million deaths and ~9 million new cases annually^1^. The global rise in these figures has begun to turn thanks to effective drug treatments and increased drive for diagnosis and treatment of infected individuals. However, the effective treatment of this disease relies upon anti-tubercular drugs in use since the 1950 s^2^ with only 1 new drug being licenced by the FDA in almost 40 years^3^. In the light of this slow introduction of new TB therapies - drug resistance, including multi-drug resistant TB (MDR-TB), has emerged as a major threat to the success of global TB management. As evidence of this, in 2013 there were an estimated 480 000 new cases of, and 210 000 deaths from, MDR-TB.

Exposure of *M. tuberculosis* to sub-lethal drug concentrations allows the bacterium to persist and, importantly, replicate in the presence of drug. Whilst the Darwinian selection of resistant mutants by lethal concentrations has received much attention over the years, there is now a growing awareness that sub-lethal drug exposure can bring about non-lethal selection and have large effects on bacterial biology^4^. Sub-lethal concentrations of anti-tubercular drugs can result from poor adherence to treatment schedules, incorrect dosing or drug regimen, irregular availability of drugs or poor penetration of the infected tissue by the drug. In illustration of this last mechanism, Dartois and Barry used mass spectrometry to demonstrate the poor penetration of human granulomas by rifampicin^5^.

High pressure liquid chromatography and modern mass spectrometry-based proteomics enables the rapid quantitation of thousands of proteins across many samples. This allows investigation, in an untargeted manner, into how the proteome as a whole changes in an organism in response to stimulus. Rifampicin binds to the RNA polymerase subunit RpoB and is one of the frontline anti-tubercular drugs; acquisition of resistance to rifampicin is a strong predictor for later development of MDR-TB^6^. Additionally, whilst one traditionally thinks of antimicrobial agents as foreign to the bacterial target, many antibiotic drugs, including rifampicin, are polyketides – a class of compound synthesised by bacteria and commonly employed in bacterial chemical warfare as a species seeks dominance in a nutrient rich environment. With this in mind, we reasoned that polyketide antibiotics such as rifampicin may in fact not be totally foreign to mycobacterial species and that ancient, broad specificity evolutionary mechanisms might therefore exist for mycobacteria to sense polyketide exposure and rapidly induce phenotypic resistance.

We further reasoned that the underlying mechanisms of phenotypic resistance to rifampicin might thus be revealed through proteomic analysis of mycobacterial spp. exposed to sub-lethal drug doses. Previously, Hu *et al*.^7^ used quantitative proteomics to study mycobacterial response to isoniazid in an *M. bovis* BCG strain, whilst Koul *et al*.^8^ studied the delayed *M. tuberculosis* response to bedaquiline. Just recently de Keijzer *et al*. reported a time-course, phospho-proteomic analysis of the effects of very high dose rifampicin treatment in *M. tuberculosis*^9^. This analysis provides good insight into the early, phenotypic responses of *M. tuberculosis* to strong rifampicin challenge. As rifampicin concentration in plasma experiences pulses of high concentration, followed by fading of the same, this may be useful and complementary to the present study where we consider the phenotypic adaptations of mycobacteria exposed to a relatively low, or sub-inhibitory/sub-lethal dosage which, for the reasons discussed above, may better represent the challenge experienced by the organism *in situ*. It is also more likely that signalling responses specific to rifampicin will be uncovered in a low-dose model than in a high-dose model where general stress response is likely to overpower the specific signalling response. The time-course used in the present study also examines later time points, relative to the generation-time of the mycobacterial species, allowing the examination of time points corresponding with early response, onset of bacteriostasis and early recovery of normal growth.

Here, we used *M. smegmatis* as a non-pathogenic model for *M. tuberculosis*, since it is considered to be a suitable model for the study of regulatory mechanisms of mycobacterial resistance^10^. In the current study we report the time-dependent, quantitative dysregulation of the *M. smegmatis* proteome upon exposure *in vitro* to sub-lethal rifampicin concentrations. Through this study, we gain insight into a series of rapid, temporal changes in the *M. smegmatis* proteome that collectively enable the bacilli to avoid cell death upon rifampicin exposure and instead to become phenotypically resistant to the antibiotic.

## Materials and Methods

### Culture Conditions, Colony Counts and Killing Curves

Wild Type *Mycobacterium smegmatis* (strain mc^2^155) glycerol stocks were streaked on 7H10 Middlebrook agar plates and single colonies were picked and inoculated in 7H9 Middlebrook broth supplemented with 10% OADC Enrichment Media and 0.2% glycerol and grown to A_600nm_ 0.6 or A_600nm_ 1.2 for synchronising initial inoculum in larger cultures. Cultures were grown to mid-log phase at 37 °C on a shaker at 120–130 rpm before inoculation into 150 mL 7H9, prepared as above with the addition of filter sterilised (0.2 µm cellulose acetate filter) Tween 20 to final concentration of 0.1% to prevent clumping of bacteria.

For determination of sub-lethal rifampicin drug concentration, triplicate flasks of 150 mL liquid cultures at mid-log phase (generated as above; A_600nm_ 1.2) were treated with various concentrations of rifampicin dissolved in DMSO, or with DMSO only as a control. Cell density, and hence replication and death, were inferred from A_600nm_ readings measured at 15 minute intervals for 2 hours, followed by 30 minute intervals for a further 2 hours (4 hours total monitoring) with a final measurement approximately 14 hours later using a Varian Cary 50 UV-visible spectrophotometer. At the highest concentrations used, rifampicin showed negligible absorbance and DMSO negligible impact on cell growth.

Colony counts for mid-log phase, A_600nm_ 1.2, were determined using 7H10 agar plates and serial, ten-fold dilutions of bacteria with culture medium.

### Cell Lysis and Protein Purification

Bacterial cultures were transferred to 50 mL centrifuge tubes and bacteria harvested by means of centrifugation at 4000 rpm and 4 °C (Heraeus Megafuge 1.0 R) for 10 minutes. The supernatant was discarded, the cells washed by resuspension in PBS and the process repeated for a second wash before snap freezing cells in liquid nitrogen and storing at −80 °C.

Frozen cell pellets were subsequently thawed by the addition of 700 μL lysis buffer consisting of 1.5% deoxycholate, 1% sodium dodecasulphate (SDS), 375 μg lysozyme, 1 tablet PhosSTOP and 1 tablet cOmplete ULTRA protease inhibitor (Roche) in 10 mL 500 mM Tris-HCl, pH 7. Once thawed the bacteria were subjected to 8 rounds of probe sonication (VirSonic UltraSonic Cell Disrupter 100 at setting 10) for 20–30 seconds, with several minutes on ice in between rounds, adapted from Chopra *et al*.^11^. The lysate was clarified by centrifugation and the supernatant was purified for protein by means of methanol/chloroform precipitation. Briefly, equal parts lysate and methanol and ¾ parts chloroform were mixed and centrifuged at 4000 rpm for 5 min. A white band of protein developed at the interface and the top layer was carefully removed before the addition of a further ¾ parts (of original lysate volume) methanol. Centrifugation caused the precipitated protein band to pellet and the remaining aqueous layer was removed.

### In-Solution Digestion of Proteins and Clean-Up of Peptides

The protein pellet was solubilised by means of 6 M Urea, 2 M Thiourea in 10 mM Tris-HCl, pH 8. Bulk protein quantitation was done by means of a modified Bradford assay^12^ and digestion by means of the in-solution method reported by Borchert *et al*.^13^. Briefly, proteins were reduced by 1 mM dithiothreitol, alkylated with 5.5 mM iodoacetamide and, pre-digested with Lys-C (1:100 mass ratio) before dilution of the denaturing buffer with 4 volumes 20 mM ammonium bicarbonate. The diluted sample was then digested with Trypsin (1:50 mass ratio) overnight. Resulting peptides were desalted using STop And Go Extraction tips^14^ (STAGE tips) and solubilised in 2% acetonitrile in preparation for LC-MS/MS.

### LC-MS/MS

Samples were fractionated in-line by means of a Dionex Ultimate 3500 RSLC Nano System (Thermo Fisher Scientific) running a reversed phase gradient over an in-house built 40 cm column (75 μm internal diameter; 3.6 μm Aeris Peptide C18 beads, Phenomenex 04A-4507) and maintained at 40 °C. Solvent A was 0.1% Formic Acid in HPLC grade water and solvent B 0.1% Formic Acid in Acetonitrile. Gradient consisted of holding 1% solvent B for 10 minutes, increasing to 6% B over 2 minutes and then increasing to 35% B over 118 minutes; washing with 80% B followed.

Tandem mass spectrometry analysis was performed using a Q-Exactive mass spectrometer (Thermo Fisher Scientific) operating in top 10 data-dependant acquisition mode. Precursor MS^1^ scan range was between 300 and 1,750 with resolution of 70,000, and automatic gain control (AGC) target of 3e6 and maximum fill time of 250 ms. Fragmentation of precursor ions was set to a normalised collision energy of 28. MS^2^ scans employed a resolution of 17,500 and an isolation window 2 Th. Scan range for MS^2^ was 200 to 2,000 Th, AGC target was set to 5e4 and maximum fill time was 80 ms. Sample injection volumes were adjusted so as to yield a total ion count of approximately 5e9 at the highest point in the peptide region for each sample.

### Protein Identification/Quantitation

Raw data files from the Q-Exactive were processed in MaxQuant^15^ version 1.5.0.3. The *M. smegmatis* mc^2^155 reference proteome from Uniprot^16^ (6,600 entries) was used to define the search space for the built-in Andromeda search engine^17^. Methionine oxidation and N-terminal acetylation were set as variable modifications and carbamidomethylation of cysteine as a constant modification. The data mass measurement corrections were performed by means of a first search with 20 ppm accuracy tolerance which was followed by a main search on the re-calibrated data with 4.5 ppm tolerance. Missed cleavages were limited to at-most two and an empirically derived false discovery rate (FDR) of 1%, estimated using the reversed proteome in a target-decoy approach, was used to restrict identifications at both the peptide spectrum matching and protein inference levels. Protein inference required at least one unique or razor peptide for identification of a protein group. The label free quantitation (LFQ) was enabled through the MaxLFQ algorithm^18^. The mass spectrometry proteomics data have been deposited to the ProteomeXchange Consortium^19^ via the PRIDE^20^ partner repository with the dataset identifier PXD004197.

### Data Handling and Statistical Treatment

The resulting protein quantifications were batch normalised using R^21^ and Combat^22^ (contained in the SVA package^23^), outlier samples were identified by means of hierarchical clustering and principle components analysis plots and were repeated. Subsequent data processing was done using Perseus (www.perseus-framework.org) and R: protein identifications were filtered *in silico* so as to consider only proteins with non-zero LFQ values for triplicate measures and mean values for each protein were compared between treated and control groups using a student’s t-test (assuming equal variance) with a cut-off value of p = 0.05. A second level of stringency was used for initial analysis wherein the absolute value of a protein’s log-2 transformed fold-change was required to be greater than twice the standard deviation of the same for all proteins in a given time-point. Those proteins passing the second criteria were referred to as being strongly dysregulated. Within each time point, those proteins entirely absent from either treatment group, whilst consistently observed in the other, were also considered to be dysregulated. Any proteins discussed in the text that were identified through presence/absence are explicitly labelled as having been so identified.

### Gene Ontology Analysis

STRING^24^ (http://string-db.org/) and the STRINGdb^25^ package in R were used for Gene Ontology terms enrichment analysis. The full set of identified mycobacterial proteins was used as the background proteome by which to calculate enrichment. The resulting enriched terms were filtered for those containing fewer than 100 proteins so as to display fewer overly-general terms.

## Results

### Determination of Sub-Lethal Rifampicin Concentration

**Figure 1:**
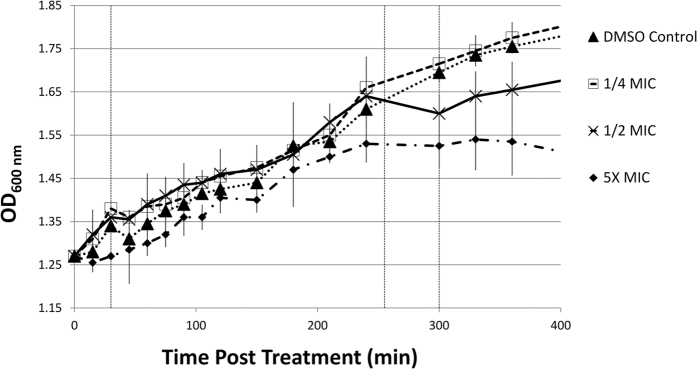
Growth Curves for cultures treated with various rifampicin concentrations at mid-log phase. MIC was 20 μg/mL and so 5X, 1/2 and 1/4 MIC indicate cultures treated with rifampicin at 100, 10 and 5 μg/mL respectively. DMSO control was treated with DMSO only. 1/4 MIC showed no difference in growth relative to DMSO control. 1/2 MIC onward showed growth defect relative to controls from 240 minutes until recovery after 300 minutes excepting 5X MIC which showed no signs of recovery. 1/2 MIC, 10 μg/mL, was selected for use as a sub-lethal concentration. Vertical dashed lines indicate time points 30, 255 and 300 minutes post-treatment which were used for time course experiment. Time points correspond to initial response, onset of bacteriostasis and early recovery respectively. Error bars indicate standard deviation.

Figure 1 shows the various growth curves obtained for different concentrations of rifampicin. Bacteria were treated with various concentrations of rifampicin at mid-log phase which corresponded to A_600nm_ of 1.2. Serial dilution plate counts determined this to correspond to 3.8e8 CFU/mL. The Minimal Inhibitory Concentration (MIC) was reported in the literature^26^ to be 20 μg/mL and this was confirmed by visual MIC assays. Various multiples of this concentration, including 1/4X, 1/2X, 1X, 2X and 5X this MIC were tested for growth effects through growth curves (1X and 2X in Supplementary Fig. S1). 5X MIC (100 μg/mL) resulted in a slight and immediate growth defect whilst all lower concentrations initially displayed growth similar to that of the DMSO only control until 240 minutes whereafter growth retardation was evident for all excepting the 1/4 MIC treated culture which showed no difference in growth relative to the DMSO control. After 300 minutes the 2X and 5X MIC (40, 100 μg/mL) concentrations appeared bactericidal whilst the 1/2 MIC and 1X MIC (10, 20 μg/mL) treated cultures showed recovery of growth after 300 minutes and optical densities rose parallel to that of the DMSO controls, only delayed. It was thus concluded that 1/2 MIC (10 μg/mL) induced bacteriostasis between 240 and 300 minutes post-treatment, with recovery evident after 300 minutes post-treatment. Thus the time points selected for proteomic investigations were 30, 255 and 300 minutes post-exposure corresponding with early exposure, onset of bacteriostasis and just prior to recovery for 1/2 MIC (10 μg/mL) – which was selected as the sub-lethal rifampicin concentration for this study.

### Data Quality

**Figure 2:**
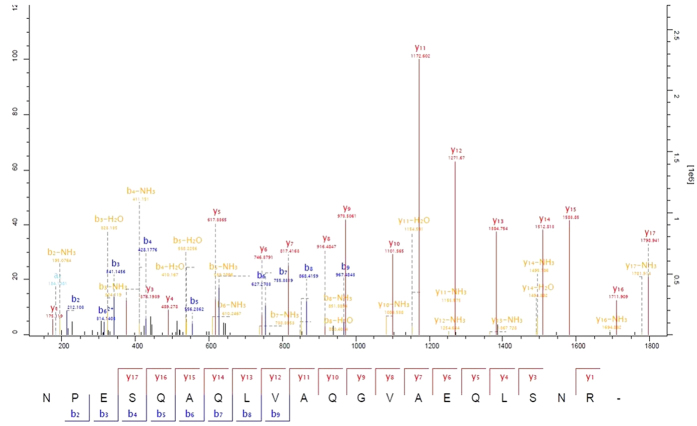
Example of an annotated MS2 spectrum. An exemplary spectrum showing high coverage of the matching peptide sequence and annotation of a high proportion of spectrum peaks.

Across 18 samples, using in-line reverse-phase chromatography of unfractionated samples, a total of 3,218 proteins were identified from 31,846 distinct peptides averaging 16,250 identified peptides per sample (see Supplementary Fig. S2), and 10 unique peptides per protein, with a total of 997,139 spectra submitted with high accuracy (average mass deviations less than 1 ppm). Figure 2 shows an example of the collected spectra annotated with b- and y-ions from the Andromeda search results.

Batch effects due to certain replicates having been processed separately were identified and batch correction applied through the ComBat algorithm^22^ found in the SVA package^23^ in R, after which samples did not cluster by batch (replicate number) as shown in Supplementary Fig. S3. Principle component 1, which accounts for the majority of the variation in the data, appeared to separate the data primarily on treatment group and this separation was larger at more advanced time points, or rather, the level of variation observed within time points, as pertaining to those proteins weighted most heavily in the PCA analysis, enlarged with later time points.

Those proteins that met the necessary criteria for application of the t-test (i.e. non-zero values for all three replicates within treatment groups) for each time point numbered 1,843; 1,951 and 1,972 for time points one, two, and three respectively. In accordance with the normalizing assumption that overall protein abundance should not change: the average log_2_-fold change was close to zero and the standard deviation thereof varied between 0.22 and 0.30. Thus total variation was similar between samples, whilst variation due to treatment increased with time of exposure.

**Figure 3:**
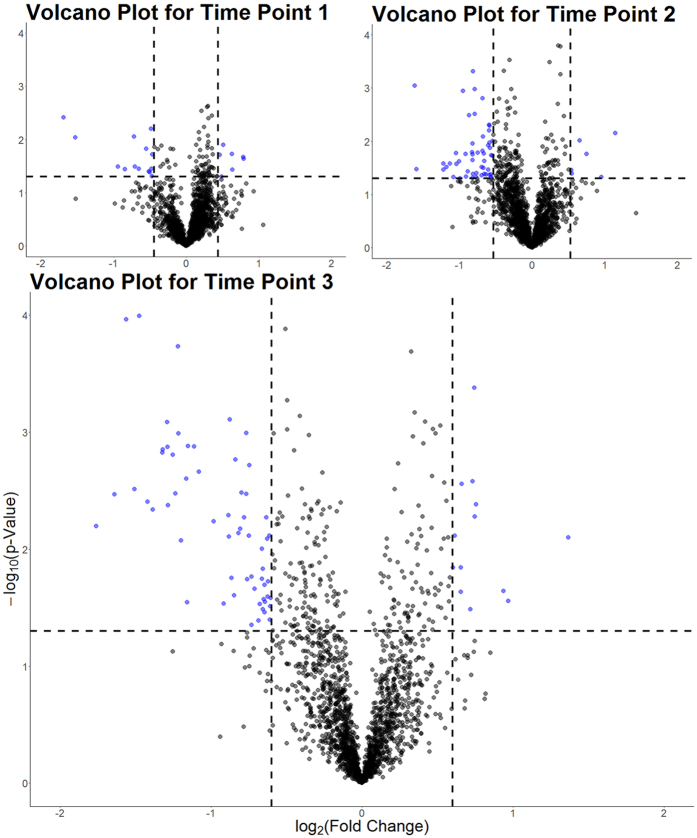
Volcano plots for time points 1, 2 and 3. Coloured points indicate those proteins significantly (p < 0.05, indicated by the horizontal dashed line) dysregulated by twice or more the standard deviation of the average fold changes for that time point (vertical dashed lines). Upper left and right panels display plots for time points one and two respectively with lower panel showing the volcano plot for the third time point.

In keeping with this observation, there were 91, 205 and 296 dysregulated (p < 0.05) and 21, 57 and 75 strongly dysregulated (by more than twice the standard deviation for fold changes within that time point; p < 0.05) proteins for time points one, two and three respectively (see Fig. 3). The 10 most strongly dysregulated proteins for each time point can be seen in Table 1. More detail is available in Supplementary Spreadsheet S1, including a list of those proteins identified as dysregulated through a presence/absence analysis in which 17, 9 and 9 proteins were uniquely expressed in either treated or control samples for time points one, two and three respectively with 15/17, 6/9 and 4/9 respectively of those dysregulated proteins being downregulated (absent in treated samples).

### Time Point 1: Initial Rifampicin Exposure is Characterised by Transient SOS Response

From the GO term enrichment analysis (see Supplementary Fig. S4) “SOS response” was, in the first time point, one of the most highly enriched biological processes with the DNA repair proteins UvrB, UvrC and RuvB all up regulated at this time point and related terms featuring in all four enrichment categories. The redox related Superoxide dismutase (Sod) was also up regulated (p < 0.05) in the first time point, consistent with reports that rifampicin causes metal catalysed ROS production^27^.

The SOS response was not found to be an enriched term in subsequent time points as significance of dysregulation for some proteins was lost. Exceptions to this were LexA, a repressor of the SOS DNA damage response, which was down regulated in the second time point, and UvrC that was observed to be again up regulated in the third time point. Thus it would seem that a transient SOS response was induced – possibly in response to oxidative stresses – and then mostly, but not entirely, silenced. Among the top 15 enriched KEGG pathways “Two-component system” features in all three time points with RegX3, PrrB, PstS and PhoP all dysregulated as early as the second time point; indicating the bacteria monitor and respond to their environment throughout the time course. It would thus appear the bacteria engage in careful sensing of the environment and, in the case of sub-lethal challenge, determine not to commit to dormancy whereas this would be expected with high dose rifampicin challenge^9^.

**Figure 4:**
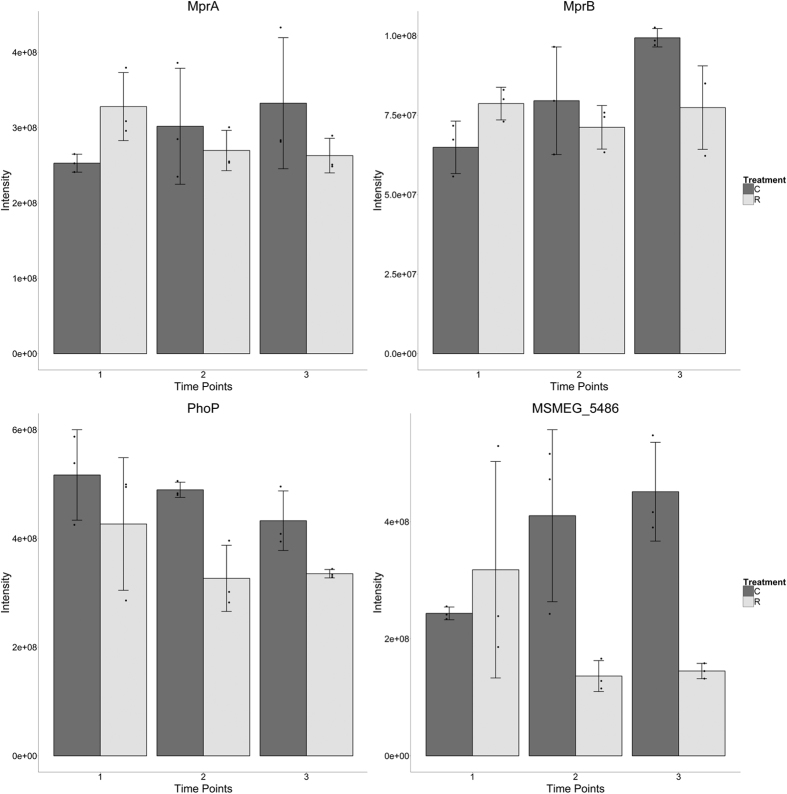
Protein expression profiles for several DevR related proteins. Upper panel, effector (left) and sensor (right) proteins of two-component system MprAB coregulate dormancy alongside DevR. Lower panel, MSMEG_5486 (left) is an orthologue of the virulence related Mtb PepD protease. PhoP (right) of two-component system PhoPR is thought to be able to modulate the DevR stress response. Bars indicate mean expression with error bars indicating standard deviation and points indicating individual replicate values. Control samples shown in dark grey and rifampicin treated in light grey.

Important persistence regulating transcription factor^28^ MprA was also observed to be significantly (p < 0.05) up regulated in the first time point (see Fig. 4). MprA has been shown to protect against various perturbants of the cell wall including cell wall antibiotics^29^ and its up regulation deserves further discussion. External to the cell wall is another important virulence factor, namely the encasing polysaccharide capsule thought to help prevent association of *M. tuberculosis* with macrophages^30^,^31^. Capsule organisation was an enriched biological process in this early time point with DprE2 and galactofuranosyl transferase GlfT2, involved in creation of the arabinan and galactan of the arabinogalactan component of the cell wall respectively, each down regulated. Both Ms3580, a predicted mycolyltransferase, and FbpB a known antigen 85-C orthologue responsible for synthesising the virulence associated trehalose dimycolate (TDM - or chord factor) from trehalose monomycolate in *M. tuberculosis*, were also observed to be down regulated in time point 1. Thus there is clear evidence for an immediate dysregulation in important cell wall and capsule organising enzymes.

In the second and third time points several enzymes involved in phosphatidylinositol mannoside (PIM) and mycolic acid synthesis, two of the major lipid forms in the mycobacterial cell walls and essential for growth, are down regulated. This could be due to decreased demand for production of such lipids due to decreased cellular replication.

### Time Point 2: Rifampicin Induces Dysregulation of DosR Response, TetR and MarR Transcriptional Regulators and Haeme Synthesis

#### DosR Response

**Figure 5:**
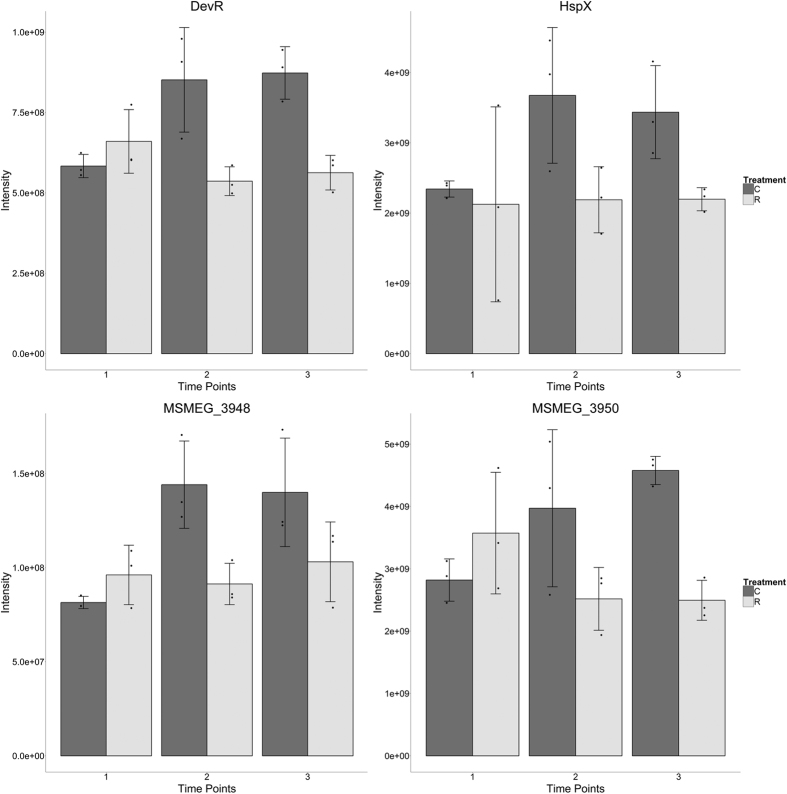
Expression profiles for representative members of the DevR regulon. Upper left panel: DevR (A0R2V2) is a major regulator in the dormancy response and is sensitive to oxidative and other stresses through DevS and DevT. Upper right panel: HspX is a stress response protein under the (activating) regulation of DevR. Lower left panel: Ms3950 is a universal stress response protein. Lower right panel: Ms3948 is an acyl-transferase. Points indicate individual replicate values measured, bars indicate mean expression with error bars indicating standard deviation. Control samples shown in dark grey and rifampicin treated in light grey.

Following on from the up regulation of MprA, the DevR (DosR) regulon was observed to be down regulated relative to controls from the second time point onward: with 13 of 33 genes known to be part of the regulon identified as dysregulated in at least one time point and all following similar expression profiles across time points. Figure 5 shows the expression profiles for 4 proteins in the regulon: DevR (the transcriptional activator of the regulon), HspX (a major stress response protein), Ms3950 (another universal stress protein; USP) and Ms3948 (an acyl-transferase representative of the non-USP proteins in the regulon). In all cases the expression in the controls rises steadily, or else rises and plateaus, whilst in the rifampicin treated cultures expression of the protein either remains constant or drops and plateaus.

#### Transcriptional regulators

In the second time point there was greater dysregulation of transcriptional regulators with 18 transcriptional regulators down regulated and just 4 up regulated. Those down regulated included five TetR family regulators, including Ms1380 identified by presence/absence, and the only MarR (Ms1492) identified as significantly dysregulated after data quality filters. TetR and MarR regulators are both typically repressors of gene transcription whose down regulation is commonly associated with multi-drug resistance and efflux pumps^32^,^33^. Accordingly, from the second to the third time points there were increasing numbers of dysregulated ABC transporters causing “ABC transporters” to appear among the top 15 enriched KEGG pathways in the second and third time points. Whilst diverse in their annotated functions and showing no obvious pattern in expression profiles, this increased dysregulation is notable as up regulation of ABC transporter drug efflux pumps is a common mechanism of MarR induced drug resistance. Also, of the 4 proteins identified as down regulated (absent in treated samples) in time point three, two are predicted transporters of the major facilitator superfamily. A recent study identified Ms4022 (MSMEG_4022) as a novel TetR-family regulator that directly activates the expression of seven transport related genes and enhanced *M. smegmatis* resistance to anti-tuberculosis drugs including rifampicin^34^. In the current study however Ms4022 did not significantly differ between treatment and control groups across all three time points (see Supplementary Spreadsheet S1). Additionally, we have identified five of the related proteins of which four (Ms1448; Ms2727; Ms5278 and Ms6909) were not significantly different and one was down regulated (Ms5051); suggesting that under our experimental conditions *M. smegmatis* employed different transport resistance mechanisms in response to rifampicin challenge.

#### Haeme Synthesis

GltD and GltX feed into the “Porphyrin and chlorophyll synthesis” pathway which was found in the top 15 enriched KEGG pathways of time point two. The iron-dependant repressor IdeR was down regulated in the second time point with iron scavenging mycobactin synthases MbtA and MbtE up regulated in the second and third time points – dysregulation of the mycobactins indicating that the treated bacteria were possibly attempting to increase iron uptake. Increased iron uptake and abundance of siderophores have previously been linked to success of the *M. tuberculosis* Beijing strain and rifampicin tolerance^9^,^35^,^36^. Supplementary Fig. S5 shows the pathway from glutamate to haeme synthesis was dysregulated in a coordinated fashion. At several branch points along the pathway there was dysregulation observed that would favour increased haeme production: notably chelatase Ms2615 and HemD. Ms6306 and Ms2383, each named GltX, are up regulated in the second and third time points respectively (the former by presence/absence) and likely generating increased quantities of Glutamyl-tRNA^Glu^ to feed into this pathway while the down regulation of HemA may have served a conflicting purpose to increase the availability of charged tRNA for translation.

### Time Point 3: Growth Recovery is Preceded by Up Regulation of Transcriptional and Translational Machinery and Mediated by Drug Inactivation

#### Redox

By time point three, both the dehydrogenase type 2a subunits, HybA and HybC, and almost the full complement of dehydrogenase-2 accessory/chaperone proteins, HypB-HypF, were down regulated. This is notable as the HybA and HybC subunits are part of the [NiFe] hydrogenase Hyd1 shown to constitutively catalyse the oxidation of hydrogen and aid growth in *M. smegmatis*^37^. The down regulation of this protein and its related accessories may offer further evidence of attempts to limit metal catalysed ROS production.

#### Growth Recovery

Time point 3 represents the time at which proteomic changes that enable recovery of growth were expected to be observable, prior to actual resumption of growth. However, in terms of bacterial replication machinery, “DNA replication” remained an enriched term and there was little other evidence in the expression profiles for replicative recovery in time point three. Indeed, NrdE2 and NrdF2 of the ribonucleoside-diphosphate reductase (responsible for reducing RNA to DNA) were down regulated further in time point three and several other cell division related proteins, such as CwsA, FtsH and FtsZ, were also down regulated in the third time point. It would thus appear that recovery of cellular replication machinery, and replicative recovery occurs closer to the subsequent time point on the curve than anticipated (see Fig. 1).

#### Transcription/Translation

Supplementary Fig. S4 shows a strong cluster of dysregulated proteins at time point three, including both transcriptional RNA polymerase subunits and translational ribosomal subunits. Supplementary Fig. S6 shows that the expression levels of RpoA, RpoB and RpoC all gradually decreased in control samples across the time-course, but steadily increased in rifampicin treated samples. Expression levels of the sigma factor SigF generally increased in both treated and control samples across the time-course, but notably SigF showed a marked down regulation at the first time point in rifampicin-treated cells and did not subsequently recover to the same levels observed in the control samples at any time point.

Supplementary Fig. S7 shows a similar general trend of reduced ribosomal expression with time in the control samples, and expression in the treated samples generally increased. In the majority of cases the protein expression of the treated and control samples were approximately equal in the first time point (with RplF being the exception) but unlike with the transcriptional machinery in Supplementary Fig. S6 some ribosomal proteins did not follow a monotonically increasing pattern of expression in the treated samples. These proteins instead showed a general increase relative to the controls but with a dip in expression in time point two that rose again in the third time point. From the GO enrichment analysis “RNA polymerase” and related terms “RNA polymerase activity” and “Primosome complex” appeared among the top 15 KEGG pathways, molecular functions and cellular compartments from the second time point onward and “Ribosome” in the top 15 most enriched cellular compartment terms in the third time point along with “Translation” which appeared in the top 15 enriched biological processes in the third time point. This, together with the expression profiles of those proteins observed to be dysregulated, argues that up regulation of both transcriptional and translational machinery was necessary to overcome the inhibitory effects of rifampicin on protein production.

#### Arr

Ms1221, or rifampin ADP-ribosyl transferase, is a broad specificity enzyme that is reported to inactivate rifampicin through ADP-ribosylation on C23, with inversion of configuration at C1 of the NAD^+^ -derived ribose, and is thought to account for the larger rifampicin MIC values for *M. smegmatis* as compared with those of *M. tuberculosis*^38^. This protein was consistently missing in control samples (absent in 8 out of 9 samples) and consistently present (8 out of 9 samples) in treated cultures and, as shown in Supplementary Fig. S8, showed steadily increasing expression in the treated samples over time. It would thus appear the most straight-forward explanation for recovery of growth in *M. smegmatis* lies in the gradual accumulation of this rifampicin-deactivating enzyme.

## Discussion

A great deal of our current knowledge regarding bacterial acquired antibiotic resistance is based on assays using high or lethal concentrations of antibiotics that would necessarily favour the selection of rare mutants with strong, pre-existing phenotypes or genotypes^39^,^40^. However phenotypically resistant bacteria can also be selected at antibiotic concentrations several hundred-fold below the lethal concentration for susceptible cells^4^,^40^,^41^. This argues that the most relevant mycobacterial responses to antibiotic exposure include those observed at sub-MIC antibiotic concentrations, which are sub-lethal and allow continued growth of the pathogen^40^. Within this context, our data shed new light on numerous molecular mechanisms which contribute to the acquisition by mycobacteria of phenotypic resistance to rifampicin – including in *M. smegmatis* the eventual up regulation of an *M. smegmatis*-specific, broad specificity chromosomal ADP-ribosyl transferase – and contrast with previous studies that selected and characterised pre-existing persister cell populations at higher antimicrobial doses^9^,^42^,^43^,^44^.

### Two component signal transduction systems a primary control mechanism mediating mycobacterial adaptive responses to sub-MIC rifampicin

It has been suggested that at sub-MIC concentrations several antibiotics act as signalling molecules inducing changes associated with gene expression, quorum sensing, biofilm formation and virulence^4^. However the circumstances and respective temporal dynamics of such responses remain largely elusive. Here, we employed an *in vitro* experimental design that allowed us to examine the antibiotic-induced proteomic changes that take place in the model organism *M. smegmatis* at different time points after an initial insult using a sub-MIC concentration of rifampicin. Our analyses focused on quantifying the temporal dynamics of differential protein expression across early response, stasis and regrowth phases post-antibiotic exposure (Fig. 1), in order to gain insight into the induction of phenotypic antibiotic resistance in mycobacteria. Amongst others changes, our data revealed a differential regulation of different two-component systems across the three time points, including the MprA/MprB and LuxR systems (Figs 4 and 5). Two-component systems are well known signal transduction devices responsible for important bacterial adaptive responses to environmental stimulus, mostly by modulating gene expression^45^. Concordant with our findings, a recent study showed that several two component systems are up regulated in multidrug resistant *M. tuberculosis* clinical isolates and could be induced by first line antitubercular agents^46^. Here, we highlight the differential regulation of a stress responsive two component system, comprising a sensor kinase MprB and response regulator MprA which act as a signal transduction pair *in vitro* and *in vivo*^47^ and which are required for persistence during host infection^48^. In *M. tuberculosis* the activation of this two-component signal transduction system directly regulates the expression of sigma factors sigB and sigE - which in turn regulates pepD^48^. This is of particular interest since, according to our data, the induction of MprA at time point 1 (Fig. 4) was followed by a later (time point 3) down regulation of its cognate kinase MprB with the pattern of PepD abundance mirroring that of MprA and showing a significant and strong decrease in abundance in the latter time points (Fig. 4). Our data therefore suggest MprA/B as an important and versatile regulatory mechanism, in which the respective components can be regulated separately such that by the up regulation of the transcriptional regulator MprA enables mycobacteria to achieve a rapid response to initial rifampicin insult, whilst its activity can later be decreased by the down regulation of the sensor component MprB.

In another two-component system, the observed dysregulation of the LuxR family response regulator DevR deserves further attention. DevR-DevS/DosR-DosS is one of the best characterised two component systems of *M. tuberculosis* and responds rapidly to oxidative stress signals, including hypoxia and nitric oxide^49^,^50^,^51^, inducing the DevR dormancy programme in mycobacteria by regulating the expression of an ~48 gene regulon^49^,^52^. A recent proteomic study on *M. tuberculosis* Beijing B0/W148 exposed to high doses of rifampicin revealed the induction of DosR regulon proteins upon antibiotic exposure^9^. By contrast, our results show two LuxR family two-component response regulators (A0R2V2 and A0QZ95) being down regulated at time points 2 and 3 (A0R2V2 seen in Fig. 5). Furthermore, whilst our data provides no direct evidence for DosR induction, within our dataset we observed 13 of the 33 proteins in the DevR regulon being down regulated (Regulon as defined in the manually curated RegPrecise; http://regprecise.lbl.gov/RegPrecise/index.jsp). It is of course possible that, in our study, DosR was in fact rapidly up regulated following rifampicin insult and then down regulated again to initial baseline by time point 1. However, it is also possible that exposure of mycobacteria to sub-lethal rifampicin challenge may not induce the DevR operon. Interestingly, an earlier quantitative proteomic analysis involving two *M. bovis* strains showed that increased expression of BCG_2013 (latency protein belonging to DosR regulon) leads to a decrease in MprA expression followed by up regulation of KatG and consequently increased INH susceptibility^53^ (Hu *et al*.^7^). By contrast, in our study MprA was up regulated at time point 1, supporting the idea that sub-MIC rifampicin does not necessarily trigger a dormancy response. This is further supported by the findings of Bartek *et al*. who observed that induction of the dormancy response was not required for drug tolerance in mice^54^. It has also been shown that induction of DevR alone is not sufficient to alter growth dynamics^55^ and moreover W-Beijing lineages of *M. tuberculosis* constitutively express this regulon^56^. Overall then, our data suggest that there may be an immediate preparatory stress response by the bacteria to the initial rifampicin insult, but that the bacteria continue to monitor their environment through “Two-component system[s]” (enriched in all three time points) and other sensing mechanisms and subsequently quell any early dormancy response, allowing the later recovery of growth once phenotypic resistance has been attained.

### Sub-MIC levels of rifampicin induce SOS response and virulence determinants

Sub MIC antibiotic concentrations have been reported to increase mutagenesis, in particular for those drugs known to interfere with DNA/RNA synthesis^40^. Our data indicate that after rifampicin insult, *M. smegmatis* primarily uses an SOS system - specifically uvrABC (which is involved in nucleotide-excision repair) - as a rapid means by which to deal with possible DNA damage. Additionally the down regulation of LexA, (a repressor of SOS DNA damage response) and the up regulation of UvrD/REP family (an inhibitor of RecA- mediated DNA strand exchange^57^) implies that SOS system was in fact activated. To our knowledge this is the first report of mycobacteria associating the induction of the SOS response system with presence of sub-lethal antibiotic concentrations, despite the induction of SOS response by sub-MIC antibiotic concentrations being well established in other bacterial species (e.g. *Escherichia coli*^58^,^59^; *Staphylococcus aureus*^60^; *Vibrio cholerea*^61^). For example, in *E. coli* fluoroquinolones are known to induce an SOS response, whilst other antibiotics, such as aminoglycosides, tetracycline and chloramphenicol, do not^62^. It will therefore of interest to explore in the future which and under what conditions different antibiotics induce SOS response in mycobacteria.

There is an increasing body of evidence indicating that several antibiotics stimulate the production of reactive oxygen species in bacteria^63^,^64^ and that sub lethal antibiotic treatment leads to multi-drug resistance, through a radical based mutagenic mechanism^65^. In line with this, our data revealed an up regulation of several oxidative stress responsive proteins across all time points, including proteins such as superoxide dismutase, oxidoreductases, thioredoxin and others. In addition to the important role of such detoxification proteins during antibiotic challenge, these proteins also represent important potential virulence determinants that may play a crucial role during bacterial survival inside macrophages, in which pathogens are thought to be primarily killed by production of ROS and NO species^66^,^67^. Our data may therefore imply that, in a scenario like that described by Dartois and Barry^68^, sub-lethal concentrations of antibiotic at the site of TB disease may have the undesired effect of potentiating pathogenic mycobacteria with tools of survival and perhaps also directly enhance the evolution of acquired drug resistance.

The quantitative proteomics analyses indicate an evident differential abundance (mainly induction) of several proteins associated with iron up take and sequestration, including MbtA and MbtE. A possible explanation for this could be the fact that, as mentioned earlier, mycobacteria respond to low concentrations of rifampicin via ancestral sensing mechanisms triggered by polyketide natural products. As mentioned above, the latter are commonly employed in bacterial chemical warfare as a species seeks dominance in a natural environment^69^. Since iron is essential for bacterial growth, it is therefore reasonable to speculate that *M. smegmatis* perceives rifampicin as a signal molecule indicating the presence of other bacterial competitors, which would immediately lead to iron uptake/sequestration as means to compete and avoid imminent iron depletion. Importantly, in the context of host infection by mycobacterial pathogens, iron uptake and sequestration have been reported to be tightly linked to virulence^70^,^71^. Thus, if similar proteomic responses are observed in *M. tuberculosis* to those reported here, sub-MIC dosing of rifampicin could have multiple detrimental effects, directly inducing a more virulent mycobacterial phenotype. Further experiments on pre-rifampicin-stressed mycobacteria are now underway to test this hypothesis.

### Identification of proteomic signatures resulting from rifampicin pre-exposure

Our growth curve data shows that after 300 mins post-rifampicin challenge (corresponding to ~2 doublings of *M. smegmatis*) the bacterial cells resume growth. Taking in to account the observed changes in the *M. smegmatis* proteome throughout the time course assay, this prompted us to investigate whether re-growing cells at time point 3 preserved markers of pre-exposure to rifampicin. Interestingly, in our data, time point 3 is characterized by the dysregulation of several ABC transporters (PstS and LpqZ were up regulated while CydD and Ms0553 were down regulated, amongst others) and, whilst neither were observed to be dysregulated in our data, we observed an inverse correlation between one MarR and one of its controlled ABC transporters, supporting the findings of Zhang *et al*.^72^ who previously identified a novel marRAB operon in which up regulation of the regulated ABC transporters resulted in increased, not decreased, susceptibility of *M. smegmatis* to rifampicin. Amongst the other ABC transporters found to be up regulated at time point 3 in our data, the up regulation of proteins such as Ferric Iron-binding periplasmic protein of ABC transporter – which is also implicated in bacterial virulence^73^,^74^ – is notable.

We also observed that oxidative stress responsive protein such as oxidoreductases and thioredoxin, as well proteins involved in iron uptake (e.g. Fe III-dicitrate-binding periplasmic Lipoprotein FecB; FeS assembly protein SufD) were up regulated at time point three. Interesting here we have also detected the up regulation of a number of additional virulence factors with a known role during infection such as zinc metalloprotease^75^ and membrane-anchored mycosin^76^ trigger factor^77^. In sum, although several stress associated proteins were down-regulated at time point 3, it is evident that even shortly prior to resumption of growth cells conserve markers of rifampicin pre-exposure, suggesting that those cells are no longer “naive” and instead continue to dysregulate certain stress responsive proteins and virulence factors that may confer a longer-lasting phenotypic advantage.

It is thus tempting to speculate overall that the proteomic responses of *M. smegmatis* to sub-lethal concentrations of rifampicin observed in the present work might be part of a more ancient, conserved, broad-specificity mechanism for mycobacteria to selectively regulate, for example, ABC transporters and the chromosomal rifampicin ADP-ribosyl transferase in order to respond rapidly to exogenous polyketide exposure; further efforts to identify the possible upstream signalling processes responsible for this phenomenon are now in hand. In summary our data demonstrates that despite *M. smegmatis* having a chromosomal resistance factor the response of the organism to sub lethal rifampicin exposure is far more complex than simple up regulation of that factor. This may reflect the fact that the resistance marker is undetectable in the absence of rifampicin, so the organism perhaps requires other more rapid stress responses to provide time for phenotypic adaption. Whether chemical blockade of these specific early stress responses will sensitise mycobacteria to lower doses of rifampicin remains to be seen.

## Additional Information

**Accession Codes**: PRIDE Accession Number: PXD004197.

**How to cite this article**: Giddey, A. D. *et al*. A temporal proteome dynamics study reveals the molecular basis of induced phenotypic resistance in *Mycobacterium smegmatis* at sub-lethal rifampicin concentrations. *Sci. Rep.* **7**, 43858; doi: 10.1038/srep43858 (2017).

**Publisher's note:** Springer Nature remains neutral with regard to jurisdictional claims in published maps and institutional affiliations.

## Supplementary Material

Supplementary Figures

Supplementary Spreadsheet S1

## Figures and Tables

**Table 1:** Ten most strongly dysregulated proteins/protein group after rifampicin exposure for each time point.

Uniprot Protein ID*	Protein Name or Description	Gene Name	log2 (Fold Change)	p-Values for Fold Change	Sequence Coverage
**Time point 1**					
A0R112	Probable nicotinate-nucleotide adenylyltransferase	nadD	0.79	2.33E-02	31.9
A0R198	Clp protease	clpP	0.78	2.14E-02	69
A0R0H6	Coenzyme F420-dependent N5, N10-methylene tetrahydromethanopterin reductase-like flavin-dependent oxidoreductase	MSMEG_4389	0.64	3.67E-02	28.3
A0R3H9	Type I antifreeze protein	MSMEG_5479	−0.65	3.55E-02	42.5
A0QT09	Succinate dehydrogenase hydrophobic membrane anchor protein SdhD	sdhD	−0.70	3.22E-02	21.8
A0QWU1	LemA protein	MSMEG_3063	−0.71	8.75E-03	53.4
A0R1D5	HpcH/HpaI aldolase/citrate lyase family protein	MSMEG_4713	−0.84	3.63E-02	29
A0QTR3	TetR-family protein transcriptional regulator	MSMEG_1935	−0.94	3.26E-02	49.8
A0R316	Uncharacterized protein	MSMEG_5308	−1.52	9.14E-03	34.2
A0R722	Glucanase	MSMEG_6752	−1.69	3.82E-03	32.3
**Time Point 2**					
A0QVH8	Zinc metalloprotease Rip1	rip1	1.15	7.04E-03	11.1
A0R2B2	Major facilitator superfamily MFS_1	MSMEG_5051	−1.04	2.70E-02	12.4
A0QUN8	Hydrogenase assembly chaperone HypC/HupF	hypC	−1.05	1.69E-02	57
A0QUN1	Tetratricopeptide repeat domain protein	MSMEG_2267	−1.08	4.76E-02	59.9
A0QTK6	S30AE family protein	MSMEG_1878	−1.13	2.64E-02	50
A0QUM7	Hydrogenase-2, large subunit	hybC	−1.18	3.08E-02	58.9
A0QXA8	Cytochrome D ubiquinol oxidase subunit 1	cydA	−1.22	2.65E-02	17.2
A0QUM5	Uncharacterized protein	MSMEG_2261	−1.22	3.41E-02	81.5
A0R3I6	Peptidase S1 and S6, chymotrypsin/Hap	MSMEG_5486	−1.59	3.36E-02	65.1
A0R045	NLP/P60	MSMEG_4256	−1.62	9.01E-04	27
**Time Point 3**					
A0QUE0	Putative helicase	MSMEG_2174	1.37	7.92E-03	56.7
A0QUM7	Hydrogenase-2, large subunit	hybC	−1.32	1.50E-03	58.9
A0R368	Uncharacterized protein	MSMEG_5362	−1.39	4.59E-03	51.2
A0R2B2	Major facilitator superfamily MFS_1	MSMEG_5051	−1.42	3.94E-03	12.4
A0QUM8	Hydrogenase maturation protease	MSMEG_2264	−1.48	1.02E-04	57.7
P0CH00/P0CG99	Ribonucleoside-diphosphate reductase subunit alpha 1/alpha 2	nrdE2	−1.51	3.06E-03	78.8
A0QUN2	Rieske (2Fe-2S) region	MSMEG_2268	−1.54	6.79E-05	50.2
A0QXA8	Cytochrome D ubiquinol oxidase subunit 1	cydA	−1.56	1.08E-04	17.2
A0R3I6	Peptidase S1 and S6, chymotrypsin/Hap	MSMEG_5486	−1.64	3.41E-03	65.1
A0QTK6	S30AE family protein	MSMEG_1878	−1.76	6.38E-03	50

(*)Q-Values for Identifications were vanishingly small and reported as 0 for proteins shown.
