# Supplementary material for: A temporal proteome dynamics study reveals the molecular basis of induced phenotypic resistance in Mycobacterium smegmatis at sub-lethal rifampicin concentrations
Source: Sci Rep. 2017 Mar 6;7:43858. doi: 10.1038/srep43858 (PMC5338346; doi:10.1038/srep43858)
Supplement: Supplementary Figures [file srep43858-s1.pdf]

Title: A temporal proteome dynamics study reveals the molecular basis of induced phenotypic resistance in *Mycobacterium smegmatis* at sub-lethal Rifampicin concentrations.

Authors: Alexander D Giddey, Elise de Kock, Kehilwe Nakedi, Shaun Garnett, Andrew JM Nel, Nelson C Soares and Jonathan M Blackburn

Supporting figures information

Supplemental Figure S1

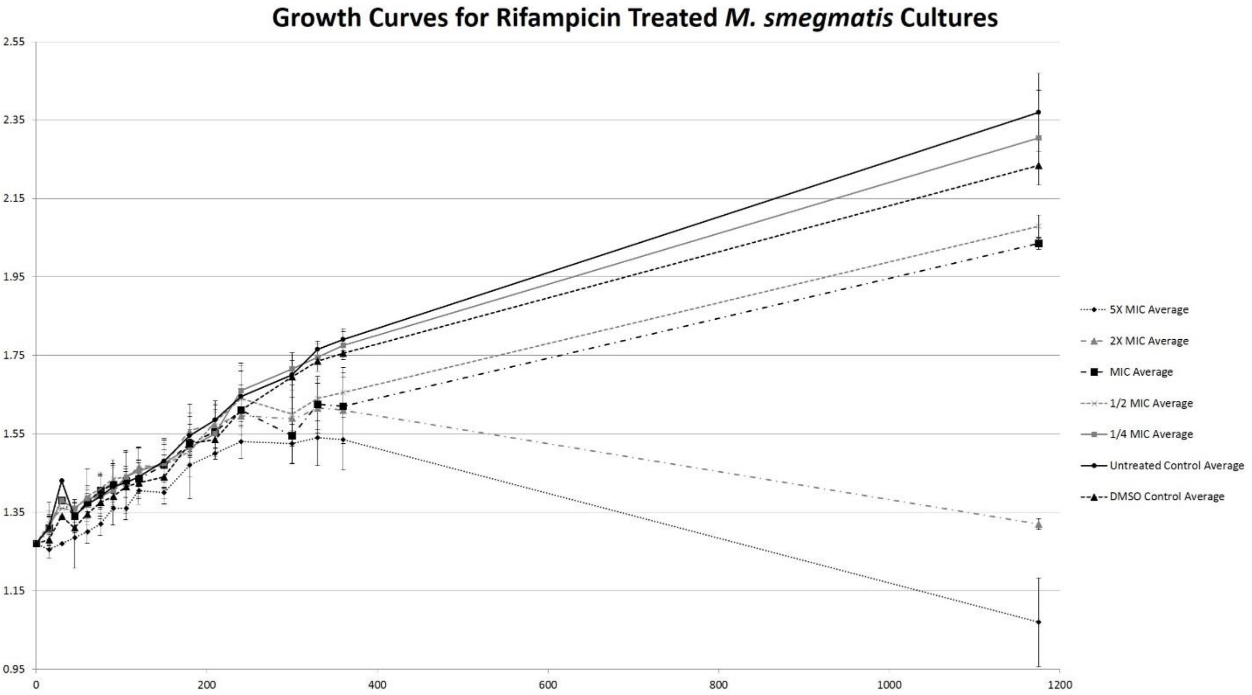

Growth Curves for cultures treated with various Rifampicin concentrations at mid-log phase.

MIC was 20 µg/mL and so 5X, 2X, 1X, ½ and ¼ MIC indicate cultures treated with Rifampicin at 100, 40, 20, 10 and 5 µg/mL respectively. DMSO control was treated with DMSO only. ¼ MIC showed no difference in growth relative to DMSO control or untreated control. ½ MIC onward showed growth defect relative to controls from 240 minutes until recovery after 300 minutes excepting 2X and 5X MIC which showed no signs of recovery. ½ MIC, 10 µg/mL, was selected for use as a sub-lethal concentration. Vertical dashed lines indicate time points 30, 255 and 300 minutes post-treatment which were used for time course experiment. Time points correspond to initial response, onset of bacteriostasis and early recovery respectively. Error bars indicate standard deviation.

Supplemental Figure S2

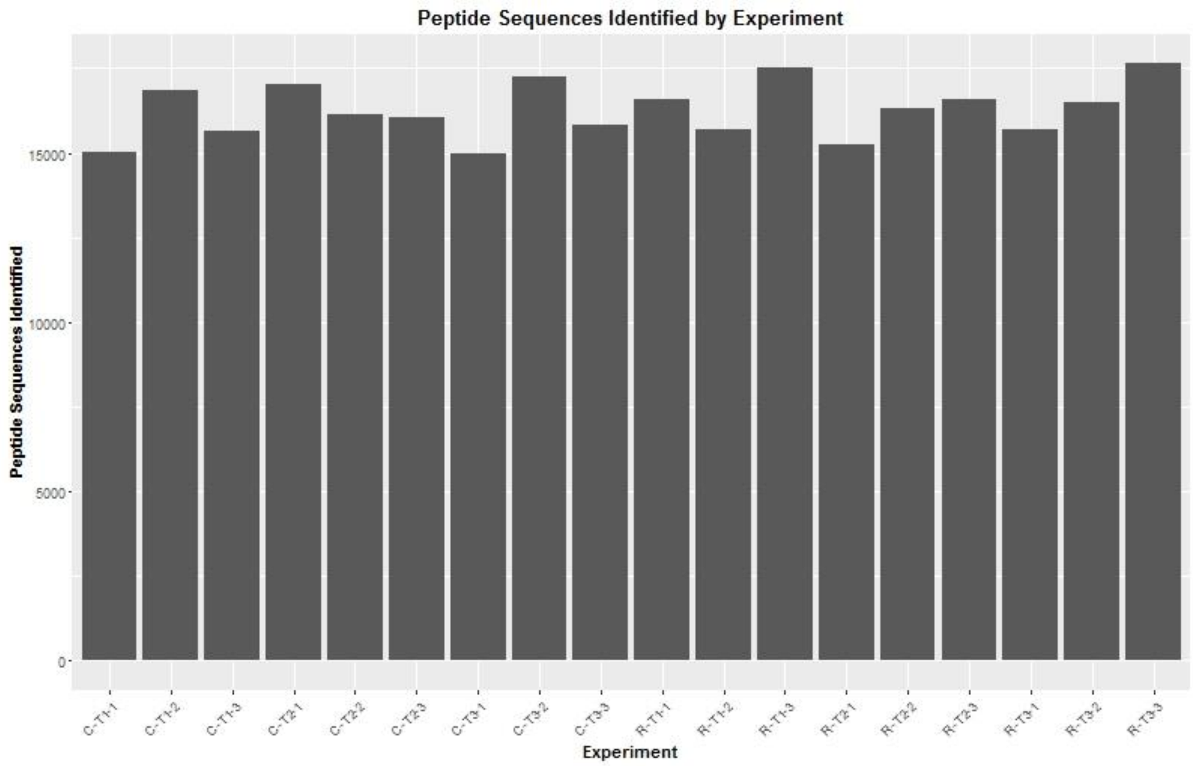

Numbers of peptides identified by sample.

C and R indicate untreated and rifampicin treated samples respectively. T1, T2 and T3 indicate time points 1, 2 and 3 respectively and the final digit indicates replicate/batch number.

Supplemental Figure S3

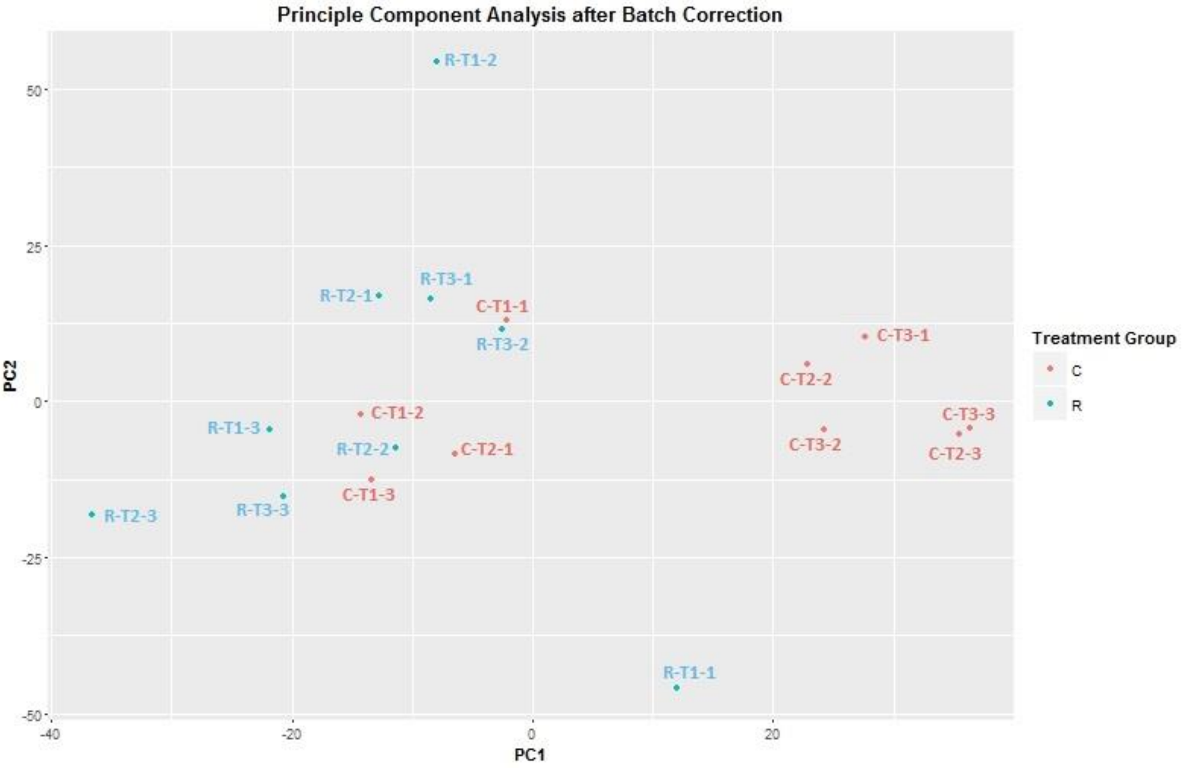

PCA plot based on LFQ values for all proteins after batch correction in R by SVA package.

Points represent samples and distances between points represents (dis)similarity between samples. Red indicates controls (C) and blue rifampicin treated (R). T1, T2 and T3 indicate time points 1, 2 and 3 respectively. Final digit distinguishes replicates/batch number.

Supplemental figure S4

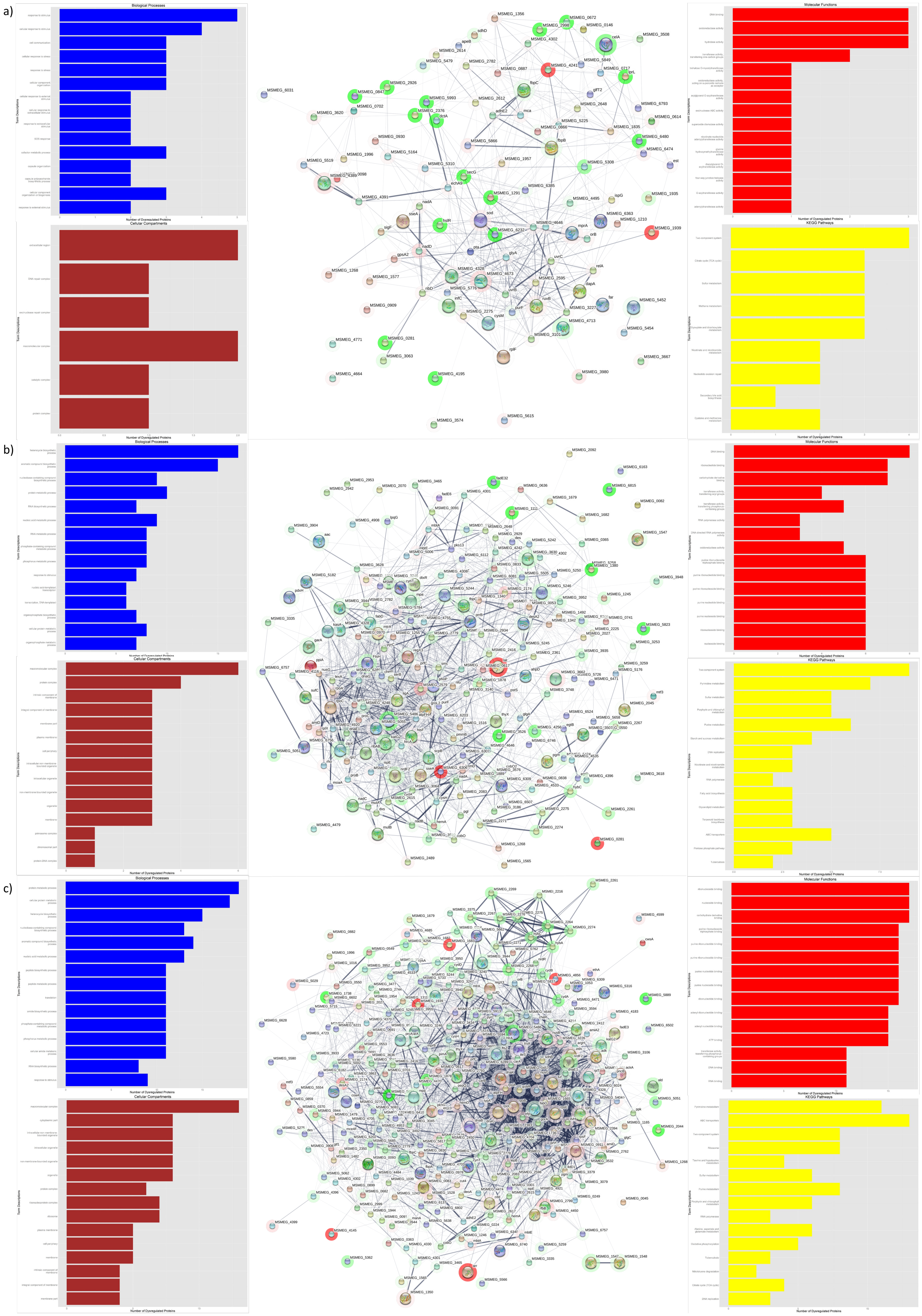

String interaction networks and gene ontology enrichment results for significantly dysregulated proteins (p<0.05)

Panels a), b) and c) represent time points one, two and three respectively. For STRING plots (center of each panel) halo indicates up/down regulation of protein at that time point, red indicates upregulation and green indicates downregulation. The thickness of the lines indicates the strength of the association/confidence in the interaction for STRING plots. Gene ontology terms ordered with smallest p-value (greatest enrichment) at the top and larger p-values to the bottom. Top 15 terms with p<0.05 after multiple testing correction shown.

Supplementary Figure S5

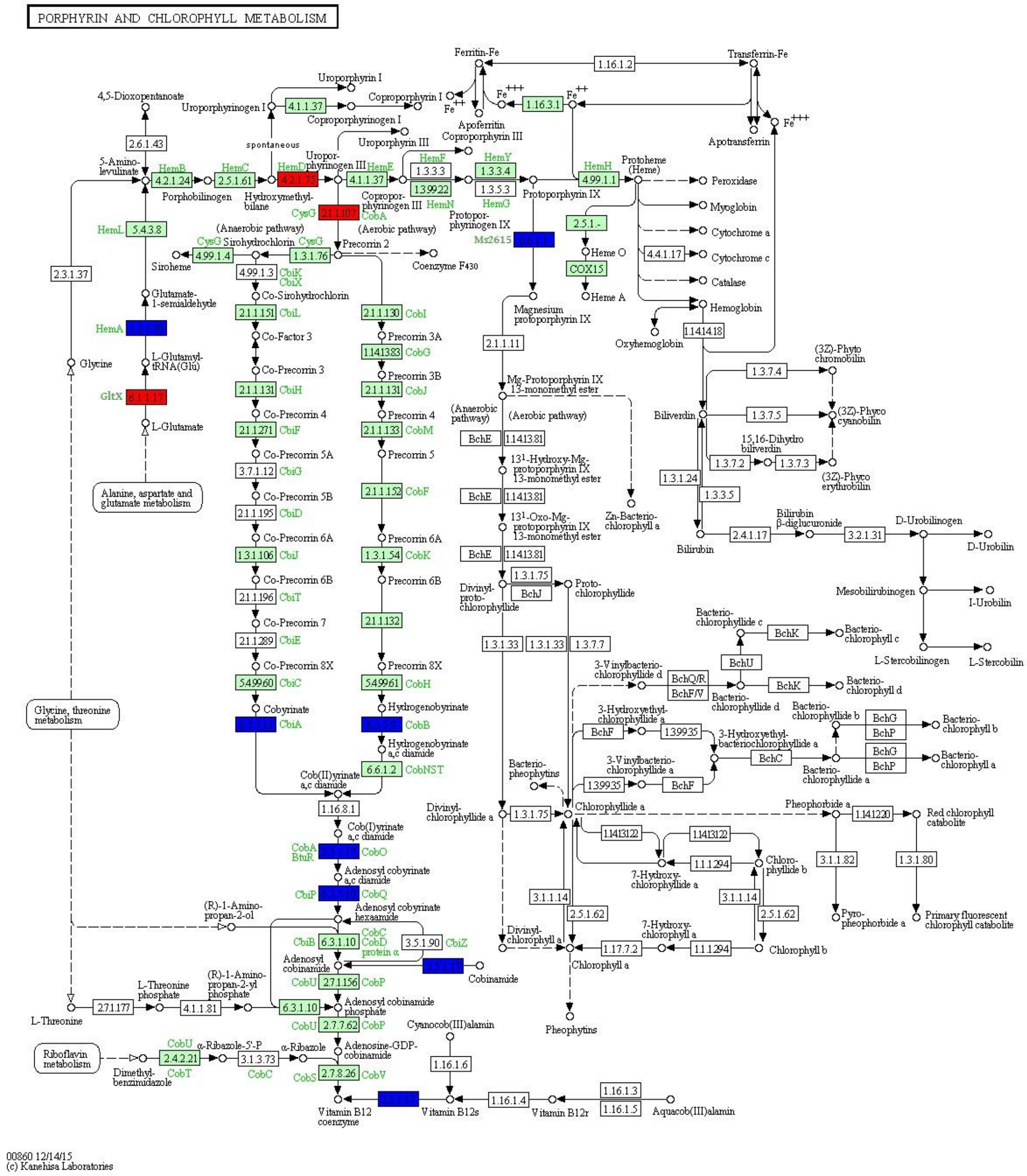

Dysregulation of glutamate path to haeme synthesis.

Blue and red coloured boxes indicate the relevant protein was down- and upregulated respectively.

Supplemental Figure S6

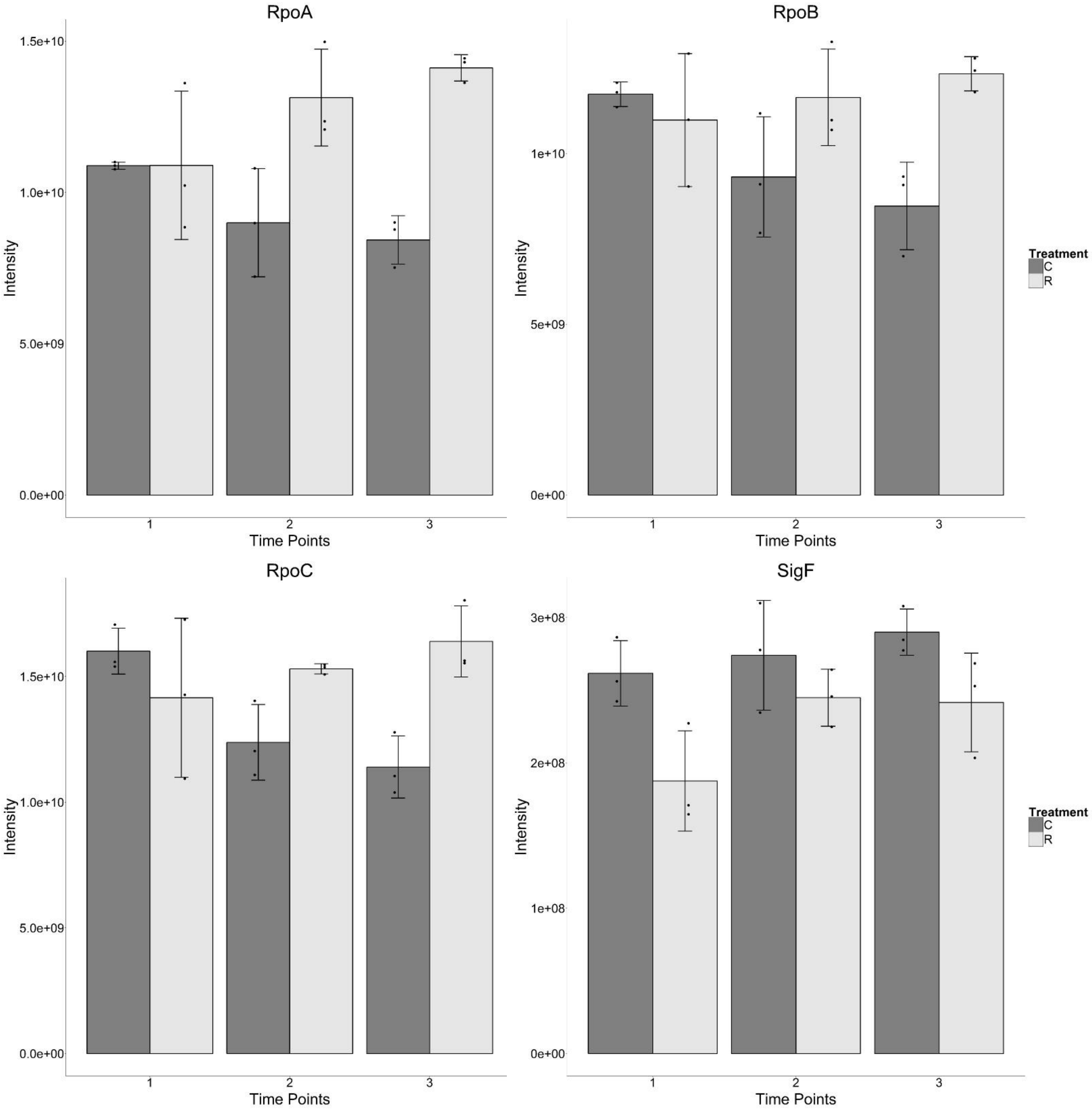

Expression profiles for RNA polymerase subunits and sigma factor SigF.

Expression profiles for RpoA (upper left panel), RpoB (upper right panel), RpoC (lower left panel) and SigF (lower right panel). Points indicate individual replicate values measured, bars indicate mean expression with error bars indicating standard deviation. Control samples shown in dark grey and Rifampicin treated in light grey.

Supplemental Figure S7

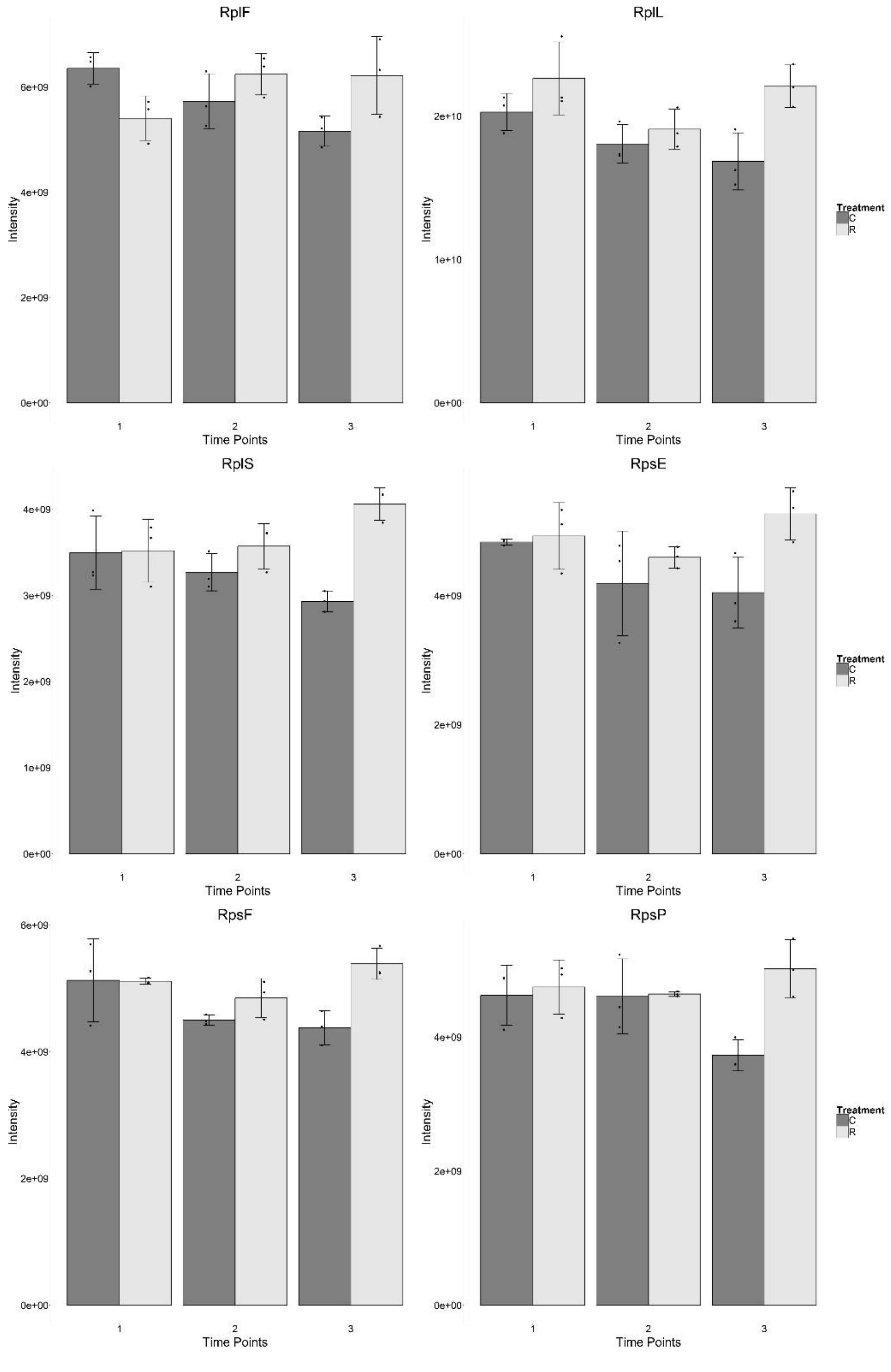

Expression profiles for representative ribosomal subunit proteins.

Expression profiles for RplF (upper left panel), RplL (upper right panel), RplS (center left panel), RpsE (center right panel), RpsF (lower left panel) and RpsP (lower right panel). Points indicate individual replicate values measured, bars indicate mean expression with error bars indicating standard deviation. Control samples shown in dark grey and Rifampicin treated in light grey.

Supplemental Figure S8

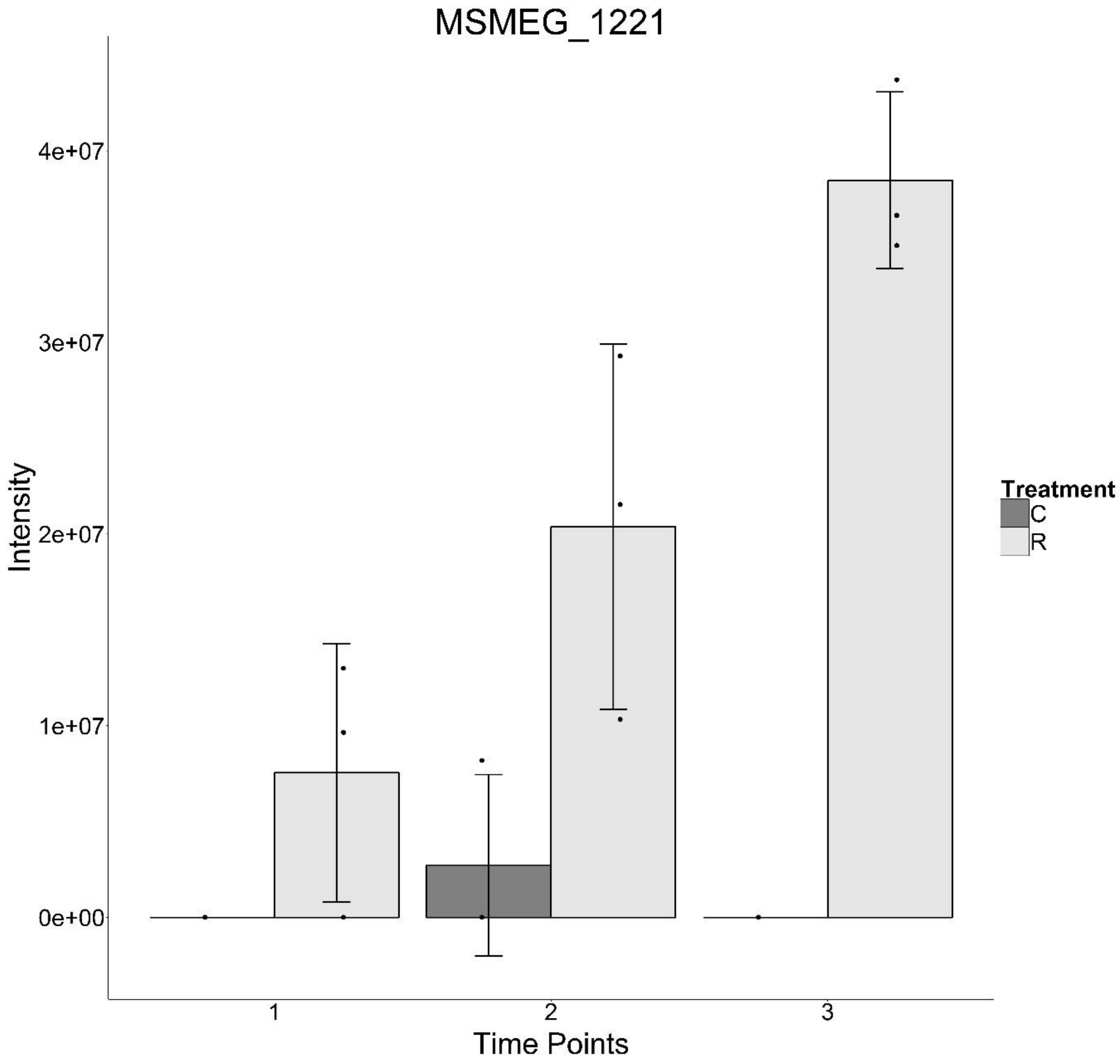

Expression profile for Ms1221, Rifampin ADP-ribosyl transferase.

Bars represent the mean of (median-normalised) raw intensity values and not mean LFQ Intensity values. Points indicate individual replicate values measured, bars indicate mean expression with error bars indicating standard deviation. Control samples shown in dark grey and Rifampicin treated in light grey.
